# COVID-19 and renal involvement in children: a retrospective study

**DOI:** 10.22088/cjim.13.0.193

**Published:** 2022

**Authors:** Hadi Sorkhi, Mohammadreza Esmaeili Dooki, Maryam Nikpour, Mohsen Mohammadi, Ali Mohammadpour-Mir, Masood Kiani, Sanaz Meherabani, Sahar Sadr Moharerpour, Morteza Alijanpour, Kazem Babazadeh, Hassan Mahmoodi-Nesheli, Mohamadreza Tabatabaie, Ahmad Tamaddoni, Mohammadreza Salehiomran, Paiam Payandeh, Iraj Mohammadzadeh, Sareh Hosseinpour

**Affiliations:** 1 Non-Communicable Pediatric Disease Research Center, Health Research Institute, Babol University of Medical Sciences, Babol, Iran; 2 Clinical Research Development Center, Amircola Children's Hospital, Babol University of Medical Sciences, Babol, Iran

**Keywords:** COVID-19, renal failure, pediatrics, kidney involvement

## Abstract

**Background:**

The New coronavirus (SARS COV-2) can cause acute respiratory disease and also multiorgan dysfunction. There is insufficient data about kidney involvement in children. So, this study was done on children with COVID-19 to evaluate nephrological involvement.

**Methods:**

All children with confirmed or suspected COVID-19 who were admitted in Children Hospital .were enrolled. They were admitted in hospital from March 2020 to July 2020. Serum Blood Urea Nitrogen (BUN), creatinine, sodium, potassium, calcium and urinalysis were evaluated. Also, glomerular filtration rate (GFR) was calculated by Schertz's formula. All patients were evaluated by chest x-ray and/or computerized tomography scanning (CTS). The data were analyzed by SPSS software and P value less than 0.05 was determined as significant.

**Results:**

Forty-seven children with confirmed or suspected COVID-19 were enrolled to this study. At admission, 23.4% and 27.7% of children with COVID-19 infection had abnormal increase in serum BUN and creatinine, respectively. Also 78.8% and 25.5% of children had GFR less than 90 and 60 ml/min /1.732, respectively. Additionally, 13/47 (27.7%) of children had abnormal urine analysis (microscopic hematuria and/or proteinuria). There wasn’t a significant relationship between pulmonary lesions and abnormal reduction of GFR (P<0/05).

**Conclusion:**

In the study, the risk of AKI (acute kidney injury) and decrease of GFR and also abnormal urinalysis is high in children with COVID-19. So, more attention for detection of kidney involvement is necessary and more conservative management for prevention of AKI and decrease of GFR are recommended.

In December 2019, the new coronavirus, named as severe acute respiratory syndrome coronavirus 2 (SARSCoV-2) was reported in Wuhan, China and has been reported in the world very soon (1, 2). According to world health organization (WHO) report, this disease has high infectious and it is defined as a pandemic disease (3). Although the virus affects adults more frequently, all age groups of children may get COVID-19. From January to March 2020, 1-5 percent of COVID -19 diagnosed patients were children (3, 4). In spite of the vast range of definition criteria for diagnosis, the children involvement range in many countries was the same (5-10). In children, fever and respiratory tract involvement (cough) are the most common symptoms (4, 6). Like adults, children with prior kidney, liver and endocrine diseases may have higher risk of severe disease (11, 12). One of the major complications of COVID -19 is kidney involvement. The results of some studies in adults have shown that acute kiddy injury (AKI) may be observed in these patients (13-15). At the time of admission, 43.9% and 26.7% of adult with COVID -19, had proteinuria and hematuria, respectively. 

Also, increased serum Blood Urea Nitrogen (BUN) and decreased glomerular filtration rate (GFR) less than 60 ml/min/1.73 were reported in 13.17% and 13.1% of patients, respectively (16). There are reports about nephrology problems in adults with COVID-19 infection however, they are rare in children. Knowledge of nephrological problems in children with COVID-19 infection can be useful in preventing and treating kidney damage caused by this virus. This study was done to evaluate kidney manifestation in children with COVID -19 (north of Iran). 

## Methods

**Study design and patients: ** This study included confirmed or suspected COVID-19 children who were referred to Children Hospital in. from March 4, 2020 to July 10, 2020. Children were defined as being over two month and under 18 years old. First, children were diagnosed on the basis of clinical characteristics and exposure history. All patients were divided into two groups: suspected and confirmed. According to COVID-19 pandemic, some authors and the national flowchart defined suspected and confirmed COVID-19 infection (17, 18). So, we use these criteria for definition of confirmed and suspected COVID-19. Inclusion criteria were children over two months and under 18 years of age, and no medical history of chronic liver disease, chronic kidney disease, neuromuscular disease, and congenital heart disease and Immune system deficiency. Indications of admission were every child with criteria signs and symptoms such as respiratory distress, diarrhea, high fever, and po intolerance. Exclusion criterion was dissatisfaction of the samples or their parents for participating in the study. 

Children were considered as confirmed cases, if they had:

1. Oropharyngeal and nasopharyngeal swab specimens positive for 2019-nCoV nucleic acid using real-time reverse-transcriptase polymerase-chain-reaction (RT-PCR) assay; or pulmonary lesion in chest x-ray or CTS that strongly indicate a COVID-19, according to the infectious disease specialist and pediatric radiologist’s reports.

Suspected children:

1) History of dry cough, chills or sore throat accompanied by shortness of breath (with or without fever) that cannot be justified by another etiological factor

2) Fever or respiratory symptoms (cough or dyspnea) with close contact with COVID -19 patient during the previous 14 days

3) A person with pneumonia who had an inappropriate clinical response despite receiving appropriate treatment

This retrospective study was approved by the Institutional Review Board of.University of Medical Sciences (ID: IR.MUBABOL.REC. 1399.184).

**Data collection**

**Evaluation of clinical characteristics: **Two experienced specialists in pediatric and clinical research (Pediatric Nephrology and health science) extracted information such as demographic variables (age, sex, place of residual, etc.), signs and symptoms, laboratory results and pharmacological treatments from medical records data.

**Evaluation of laboratory results** **and pulmonary lesions findings: **After admission, Oropharyngeal and nasopharyngeal swab specimens were obtained from all patients and sent to Caspian core research laboratory. According to the guidelines of the World Health Organization the real time polymerase chain reaction (RT-PCR) test for SARS-Cov-2 RNA was done. Intravenous blood samples were sent to the laboratory for measurement of complete blood count, C-reactive protein concentration (CRP), Erythrocyte Sedimentation Rate (ESR) and kidney function tests. To evaluate kidney function, serum BUN (mg/dl), creatinine (mg/dl) sodium (mEq/L), potassium (mEq/L) and calcium (mg/dl) were measured. Also, GFR (ml/min/1.73m2) was measured according to Schwartz formula [19]. AKI is a rapid decrease in renal function that results in the reduction of glomerular filtration, and decreased urine volume as well as elevated serum urea and creatinine presence in the sample (20). Protein, blood, glucose, white blood cell (WBC) or red blood cell (RBC) in urine was checked with urinalysis. Blood culture was also measured. All children underwent Chest x-ray or CTS and criteria such as the number of lobe involvement, the location of the involvement (one side or bilateral), predominant distribution (central or peripheral) and main presence of lesion were evaluated. If needed, ultrasonography of the abdomen and kidneys was taken. All imaging features were evaluated by one experienced pediatric radiologist and an infectious diseases specialist. 

**Statistical analysis: **Continuous variables were analyzed using median and interquartile range (IQR) values and categories with counts (percentages). All statistical tests were two-tailed at the significance level of P < 0.05.

## Results

Forty-seven children diagnosed of COVID-19 who were admitted to Amirkola Children Hospital (north of Iran) from March 4, 2020 to July 10, 2020 were enrolled to this study. Of them, 24 (51.1 %) children were identified as confirmed COVID-19 cases, and 23 (48.9%) children were defined suspected cases.

**Epidemiology and Demography:** The median of children’ age was 6 (IQR, 3, 10) years. Approximately more than half of children were boys (55.3%) and the most of them lived in urban areas (63.8%). All of them lived in of epidemic areas with COVID -19 and one- quarter of them (25.5%) had a positive history of contact with COVID -19 patients or having traveled recently (23.4) (Table 1). About 12% of them had a history of asthma, allergy or hypothyroidism. 

**Table 1:** Demographic and clinical Characteristics of 47 Children’ with COVID-19 infection

**Variable**	**N (%)**	**%**
Sex		
Girl Boy	21 26	44.7 55.3
Place of residence		
City Village	30 17	63.8 36.2
History of chronic basic diseases Yes No	6 41	12.8 87.2
Fever Yes No	42 5	89.4 10.6
Cough Yes No	13 34	37.7 72.3
Weakness Yes No	33 14	70.2 29.8
Anorexia Yes No	30 36.2	63.8 36.2
Nausea-Vomiting Yes No	18 29	38.3 61.7
Diarrhea Yes No	18 9	38.3 61.7
Abdominal pain Yes No	22 25	46.8 53.2
Rash Yes No	8 39	17 83

**Clinical symptoms and treatment: **The most clinical symptoms in children were fever (89.4%), weakness (70.2%) and anorexia (63.8%). Also these children have experienced other symptoms such as abdominal pain (46.8%), diarrhea (38.3%) nausea and vomiting (38.3%), cough (27.7.4%), sore throat (25.5%). and respiratory distress (21.3%). Additionally 8 out of 47 (17.0%) of them had rash. All of them with COVID -19 were given antibiotics (such as Ceftriaxone or Clindamycin), and if needed hydroxychloroquine sulfate, and antiviral (Kaletra) were also used for treatment. Also, children with respiratory distress received bronchodilator such as Ventolin and Oxygen support with nasal cannula. In the process of management, two children needed kidney replacement therapy (haemodialysis) for about one week, because they had GFR less than 15 ml/min /1.732. Serum creatinine and BUN returned to normal level during admission. All children were discharged in good health.

**Laboratory test results, ultrasound and pulmonary CT scan: **The medians of serum BUN (mg/dl) at the time of admission and discharging from hospital were 10.80 (IQR 8.30, 15.0) and 10.20 (IQR 7.1, 13.00), respectively. The median of serum creatinine (mg/dl) at admission and discharging times were 0.61 (IQR 0.50-0.80), 0.56 (IQR 0.50-0.64), respectively. Also at the admission time the median of GFR was 76.97 (IQR 62.41-88.18) and at the discharging time was 85.04 (IQR 76.54-106). The median of sodium (mEq/L), potassium (mEq/L) and calcium (mg/dl) are shown in table 2. 

**Table 2:** The Median and IQR of kidney function test in 47 Children’ with COVID-19 infection

**Variable** (median (IQR))	**Value ** **(at admission)**	**Value ** **(at discharge)**
Blood Urea Nitrogen in mg/dL	10.80 (8.30-15.0)	10.20(7.1-13.00)
Creatinine in mg/dL	061(0.50-0.80)	0.56 (0.50-0.64)
Sodium in mEq/L	135.0 (130.0-138.60)	136.00 (133.70-138.90)
Potassium in mEq/L	4.19 (3.67-4.50)	4.49 (3.90-4.96)
Calcium in mEq/L	8.95 (8.37-9.50)	9.00 (8.90-9.80)
Glomerular Filtration Rate in ml/min/1.73m^2^	76.97 (62.41-88.18)	84.86 (63.53-99.63)

At admission time, 11/47 (23.4%) and 13/47 (27.7%) of children had increase serum BUN and creatinine, respectively, which returned to normal level at the time of discharge. Also at admission, 12/35 (27%) and 5/47 (10.6 %) of children had lack of sodium (less than 130 mEq/L) and potassium (less than 3.5 mEq/L), respectively, which returned to the normal range at the discharge time. All of them had normal calcium (mg/dl) levels at admission and discharge time. At the admission time 13/47 (27.7%) of children had abnormal urinalysis. They had positive protein, blood and glucose in urinalysis (Table 3). 

**Table 3:** The frequency of kidney function test in 47 Children’ with COVID-19

	**N**	**%**
BUN (mg/dl )		
Normal Abnmoral (>17)	36 11	76.6 23.4
Cr (mg/dl )		
Normal Abnmoral (>0.8)	34 13	72.3 27.7
GFR		
Normal Abnmoral (<90%)	16 31	34.0 66.0
GFR		
Normal Abnmoral <60%	37 10	78.7 21.3
Na (mEq/L)		
Normal Abnormal (<130)	35 12	74.5 25.5
K (mEq/L)		
Normal Abnormal (<3.5)	42 5	89.4 10.6
Ca (mEq/L)		
Normal Abnormal (>8.5)	47 2	95.7 4.3
Urine Analysis		
Normal Abnormal Protein Glucose WBC RBC	34 13 7 5 4 8	72.3 27.7 14.9 10.6 8.5 17.0

Also, they had pyuria and microscopic hematuria. The urinalysis was returned to normal at discharge time. The findings of the present study revealed that 31/47 (78.8%) of children with COVID-19 had a GFR of less than 90 ml/min /1.732. Also 10 out of 47 (25.5%) of them had a GFR of less than 60 ml/min /1.732. (Table 3) Additionally, 38/47 (80.9%) and 28/47 (59.6%) of children had elevated CRP and ESR, respectively. However, there was no significant relationship between abnormal ESR (CI: -0.27-0.52; P=0.55) and CRP and a reduction in GFR (CI: -0.36– 0.502; P=0.71). Blood culture was negative in all patients. Assessment of Chest x-ray or chest CTS examinations showed 11 out of 47 (23.4.0%) of children had pulmonary lesions. There wasn’t a significant relationship between pulmonary lesions and abnormal reduction of GFR (P=0.40). Only one child had increased kidney size in ultrasonography evaluation.

## Discussion

The aim of this study was to evaluate kidney manifestation in children with COVID -19. This study showed that 31/47 (66%) of children with COVID-19 had GFR less than 90 ml/min/1.73m2. Also, about one quarter of children had GFR less than 60 ml/min/1.73m2. Of them, 2 cases needed replacement therapy (hemodialysis) for about one week. There were not any prior kidney diseases in these children. 

COVID-19 can cause multiorgan dysfunction in various body systems such as respiratory system, cardiovascular system, and the gastrointestinal tract. Kidney involvement has been reported in the previous studies. Although, the majority of patients with COVID-19 were presented by respiratory tract symptoms (21,22,16). Kidney involvement in our patients may be due to prolong fever, oral intake intolerance and delay admission in the pandemic area So, pre-renal azotemia and then acute tubular necrosis (ATN) may be the first major cause of a decrease in GFR. 

Although the risk of AKI (acute kidney injury) in children with COVID-19 is unknown, according to adult reports, the incidence of AKI may vary 0.9- 29% in different countries (23, 15, 24). Cheng et al reported elevation of serum BUN and creatinine in 15.5% and 14.17% of patients, respectively (16). In adult patients with COVID -19 disease, Wang l et al showed only 10.8% of patients had a decrease in GRR and also 7.2% of patients had +1 proteinuria (25). In some reports, 0.5- 7% of all cases with COVID -19 and 2.9-23% of patients admitted in ICU (Intensive care unit) had AKI, respectively [2,21,26]. The cause of acute kidney injury (AKI) was not clear but some mechanisms such as sepsis induced cytokines release, or hypoxic/hypoperfusion cellular injury, angiotensin convert enzyme 2 (ACE- 2) receptors were suggested as possible explanations. ACE- 2 receptors expresses on tubular cells and so, direct invasion of Corona virus on kidney as a target organ was suspected (27,28). ACE- 2 is a receptor in surface of kidney tubular cells and may cause prolong duration of kidney injury time (29). Cytopathic injury due to direct effect of COVID -19 on podocytes and also proximal straight tubule may be the cause of acute renal failure (27). Su et al showed two different proximal tubule injuries through kidney biopsy in light microscopy: vacuolar degeneration, and necrosis. Also, pigmented cast and granules of hemosiderin were seen. So, they suggested that hypoxia, abnormal coagulation and drug nephrotoxicity may be the plausible causes AKI (24). About 43.9% of patients with COVID-19, especially with AKI may have proteinuria (16). In our cases, about 28.6% of children had abnormal urinalysis. Five patients had proteinuria and 3 cases had microscopic hematuria. But in Li z et al, massive proteinuria was reported in 34% (59 adult) of patients on the first admission day. Of course, 66% of patients had proteinuria during staying in the hospital (30). 

In our study, there was no correlation between pulmonary lesions and decrease of GFR. Based on our knowledge, there was no study on the relation between pulmonary lesions and the reduction of GFR. Pulmonary lesions create hypoxia and can decrease GFR. Our patients were treated by hydroxychloroquine sulfate and Kaletra (lopinavir/ritonavir) for COVID-19. Also, supportive care and conservative treatment for kidney involvement were done. Of course two patients needed hemodialysis for one week. All patients were discharged with normal GFR and without any nephrological problems. This study had a retrospective design; It is suggested that future studies be conducted with cohort designs. Also, our sample size was small due to the limited number of children with COVID -19 in the region.

The risk of AKI was considerably high in our patients which means early diagnosis of COVID-19 and AKI are very important. The reason can be high and prolong fever, lack of proper fluid intake, and delay in referral to care centers. More studies are needed about nephrological problems and about the outcome of COVID-19 especially in children. 
